# Visceral leishmaniasis in an environmentally protected area in southeastern Brazil: Epidemiological and laboratory cross-sectional investigation of phlebotomine fauna, wild hosts and canine cases

**DOI:** 10.1371/journal.pntd.0005666

**Published:** 2017-07-13

**Authors:** Maria Rita Donalisio, Laís Moraes Paiz, Vanessa Gusmon da Silva, Virgínia Bodelão Richini-Pereira, Andrea Paula Bruno von Zuben, Claudio Luiz Castagna, Gabriela Motoie, Roberto Mitsuyoshi Hiramoto, José Eduardo Tolezano

**Affiliations:** 1 Department of Public Health, State University of Campinas, Campinas, São Paulo, Brazil; 2 Center for Systemic Parasitic Diseases, Adolfo Lutz Institute, São Paulo, São Paulo, Brazil; 3 Bauru II Center of Regional Laboratories, Adolfo Lutz Institute, Bauru, São Paulo, Brazil; 4 Campinas Municipal Health Department, Campinas, São Paulo, Brazil; Universidade Federal de Minas Gerais, BRAZIL

## Abstract

**Background:**

Leishmaniasis is a rapidly expanding zoonosis that shows increasing urbanization. Concern exists regarding the role of wildlife in visceral leishmaniasis (VL) transmission, due to frequent natural or anthropogenic environmental changes that facilitate contact between wildlife, humans and their pets. The municipality of Campinas, in southeastern Brazil, initially recorded VL in 2009, when the first autochthonous case was confirmed in a dog living in an upscale residential condominium, located inside an environmentally protected area (EPA). Since then, disease transmission remains restricted to dogs inhabiting two geographically contiguous condominiums within the EPA.

**Methodology/Principal findings:**

We conducted a cross-sectional study of the VL focus to investigate *Leishmania* spp. infection in domestic dogs, wild mammals and sand flies using molecular tools and recommended serological techniques. Canine seroprevalences of 1.5% and 1.2% were observed in 2013 and 2015, respectively. Six insect species, confirmed or suspected vectors or potential transmitters of *Leishmania*, were identified. Two specimens of the main *L*. (*L*.) *infantum* vector in Brazil, *Lutzomyia longipalpis*, were captured in the EPA. Natural infection by *L*. (*L*.) *infantum* was recorded in one *Expapillata firmatoi* specimen and two *Pintomyia monticola*. Natural infection by *L*. (*L*.) *infantum* and *Leishmania* subgenus *Viannia* was also detected in two white-eared opossums (*Didelphis albiventris*), a known reservoir of VL. Geographical coordinates of each sampling of infected animals were plotted on a map of the EPA, demonstrating proximity between these animals, human residences, including the dogs positive for VL, and forest areas.

**Conclusions/Significance:**

The EPA, which is inhabited by humans, has an active VL focus. The risk of establishing and maintaining disease transmission foci in similar scenarios, i.e. wild areas that undergo environmental modifications, is evident. Moreover, different epidemiological profiles of VL must be included to elaborate prevention and control measures that consider the particularities of each transmission area.

## Author summary

Leishmaniasis are neglected tropical diseases that represent a major public health problem in the world. New outbreaks have been recorded in the outskirts of some cities and phlebotomine vectors show geographic expansion, potentially associated with environmental and climate changes. We investigated a recent focus of visceral leishmaniasis (VL) in dogs from two residential condominiums located in an environmentally protected area in the municipality of Campinas, São Paulo, southeastern Brazil. The purpose of the study was to investigate *Leishmania* (*L*.) *infantum* infection, the etiological agent of the disease, in vector insects (phlebotominae), domestic (dog) and wild hosts. Antibody prevalence was determined in 1.5% and 1.2% of dogs examined in 2013 and 2015, respectively. In addition, natural infection by the VL agent was confirmed in two species of sand flies and two white-eared opossums. All positive animals were captured near human residences and in forest areas. Scenarios like these, with changes in ecosystems and the proximity of wild and domestic animals and humans, can promote the occurrence of zoonoses. The participation of wildlife in the transmission cycle of zoonoses has assumed greater importance in recent years as a result of the consolidation of the One Health concept.

## Introduction

In the last 35 years, visceral leishmaniasis (VL) has reemerged as an important public health problem in different parts of the world, with the expansion of transmission and the occurrence of new cases [1].

In the Americas, the disease occurs within a complex network of relationships involving the etiological agent, *Leishmania* (*Leishmania*) *infantum* (Synonym: *L*. *chagasi*) and its different populations: sand flies, mainly of the species *Lutzomyia longipalpis* [2]; domestic or wild vertebrates, sources of vector infection; and susceptible hosts. All of these can be present in a certain time and space, interacting with the particularities of the environment.

Although the dog is considered the main reservoir in the urban environment, it is necessary to consider the possibility of other non-human hosts participating in the maintenance of *L*. (*L*.) *infantum* in the endemic environment. These include several species belonging to different orders of wild and/or synanthropic animals, which are naturally infected in several regions and ecotypes [3].

In Brazil, until the mid-1980s VL was considered a disease of primarily wild and rural environments. From the late 1980s onward, epidemics have occurred in the urban environment, together with rapid expansion of transmission foci in hundreds of municipalities, including populous urban centers and several capitals [4–6]. The rural epidemiological pattern has been modified by increasing urbanization, while the geographic expansion of the disease has been observed in previously safe municipalities [7–10]. Expansion of the spaces occupied by *Lu*. *longipalpis* has also been verified in endemic urban environments in several regions of the country [11,12].

In the state of São Paulo, southeastern Brazil, VL transmission is also in full expansion. Over a period of 17 years, from 1997 to 2014, the presence of the vector *Lu*. *longipalpis* has been confirmed in 177 municipalities in the state, with 2,467 autochthonous human cases, 214 deaths and a mortality rate of 8.7% [13,14].

It is currently possible to recognize at least three different transmission patterns and epidemiological profiles of the disease in the state of São Paulo: the first occurs in 76 municipalities and involves both human and canine VL cases, besides the presence of the vector *Lu*. *Longipalpis*. The second occurs in 47 municipalities, seven of which without records of *Lu*. *longipalpis*, and involving only canine cases, with no record of human autochthony. Finally, a third pattern involves only human cases, which occurs in nine municipalities, three of which without reports of the vector [13].

Little is known of the dynamics of the expansion of new outbreaks, the environmental and epidemiological determinants of the disease, or the dispersion and behavior of vectors and hosts potentially involved in the different VL transmission cycles. What is known, however, is that increasing environmental degradation of natural and/or anthropogenic origin is a concern in several parts of the world.

The destruction or replacement of the original vegetal cover of a region can alter the composition and interaction of its fauna [15], which can lead to modifications and adaptations in the biology of vectors and hosts of diseases like leishmaniasis.

The initial VL focus in Campinas was identified in 2009, based on an autochthonous canine case reported in an environmentally protected area (EPA) within the municipality [16,17]. This area has undergone intense environmental changes since the 1970s, with deforestation for road and residential condominiums construction.

The epidemiological investigation conducted by the Municipal and State Health Department for the first case of canine VL (CVL) in 2009 determined an anti-*Leishmania* seroprevalence of 2.0% (4/198) in dogs and none of the 40 wild mammals captured in this occasion was positive for *L*. (*L*.) *infantum* by polymerase chain reaction (PCR). Sand flies were present in 16/85 (18.8%) residences and *Lu*. *longipalpis* was verified in three of the 16 (18.8%) houses with the presence of sand flies, being 22.2% of the females positive for *L*. (*L*.) *infantum* by PCR [17].

Since it was the first suspected case of autochthony for VL and in order to confirm the occurrence of *L*. (*L*.) *infantum* for the first time in this geographic area, at that occasion biological samples collected from dogs diagnosed positive in serological tests were sent to a reference laboratory, the Adolfo Lutz Institute (IAL), which confirms the occurrence of the parasite through isolation in culture medium and molecular tools.

From 2009 to 2016, no human cases have yet been diagnosed in Campinas and notification of canine cases remains geographically restricted to the two contiguous residential condominiums located inside the EPA.

In order to identify the different components involved in the transmission cycle of this CVL focus in Campinas, we conducted a broad epidemiological investigation involving the capture of sand flies and free-living wild animals in other areas of the EPA and integrated this research with the results of canine serological surveys.

## Methods

### Ethics statement

This research was approved by the Ethics Committee on Animal Use (ECAU) of Campinas State University (protocol no. 3296–1), the ECAU of the Adolfo Lutz Institute and the Pasteur Institute (protocol no. 01/2013) and by the Brazilian Institute of Environment and Renewable Natural Resources, through the Biodiversity Authorization and Information System (IBAMA, SISBIO, no. 42926-1/2).

### Study area

The municipality of Campinas (22°53’20” S, 47°04’40” W) has approximately one million inhabitants and is located in the southeast region of São Paulo, 100 km from the state capital. In 1993, the Mayor’s Office of the municipality of Campinas delimited an area of 223 Km^2^ in the east of the city as an EPA, located between latitudes 22°45’00” and 22°56’00” S and longitudes 46°52’30” and 47°00’00” W [15,18]. This area contains the largest number of forest fragments in the municipality, including several Atlantic Forest remnants, as well as fauna diversity of 68 species of wild mammals, including several endangered species [15,19].

Despite the cultural and biological richness, over the last few decades, the EPA has undergone significant environmental changes, with the implantation of upscale horizontal condominiums, which opened up new roads, established new inhabitants and altered the delimitation of the urban perimeters [15,17,20].

**Fig 1:**
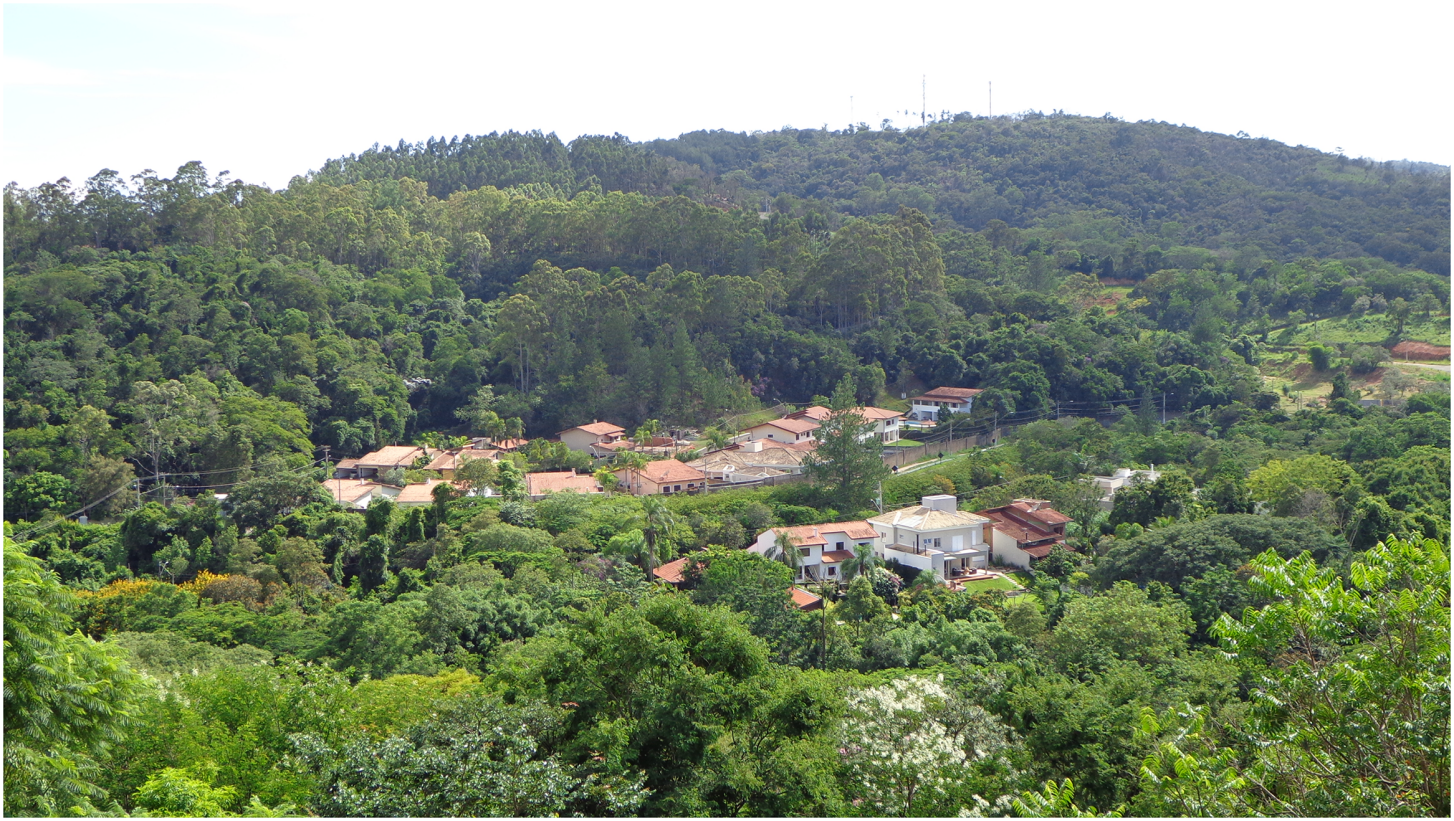
Study area. Localization of residences in the condominium where the first autochthonous case of canine visceral leishmaniasis was detected in the municipality of Campinas, São Paulo, Brazil, in 2009.

In 2009, the first autochthonous case of CVL in the municipality of Campinas was reported in a dog that lived in one of these residential condominiums inside the EPA, close to residual forest fragments, where contact between the wild fauna, humans and their pets can occur [16,17]. Since then, new canine cases have been reported in this same condominium and in another that is geographically contiguous and shares the same environmental characteristics. The proximity of human residences to these green areas is shown in Fig 1, together with the area of CVL focus in the EPA.

We set traps at 18 different points throughout the EPA to capture vectors and wild animals. These capture points were defined based on criteria that included easy of access by roads, proximity to water courses, size of the forest area, forest connections with urban perimeters and proximity to the CVL focus. They were defined with the help of Google Earth software (Google, version 7.1.2.2041, compiled in 2013).

**Fig 2:**
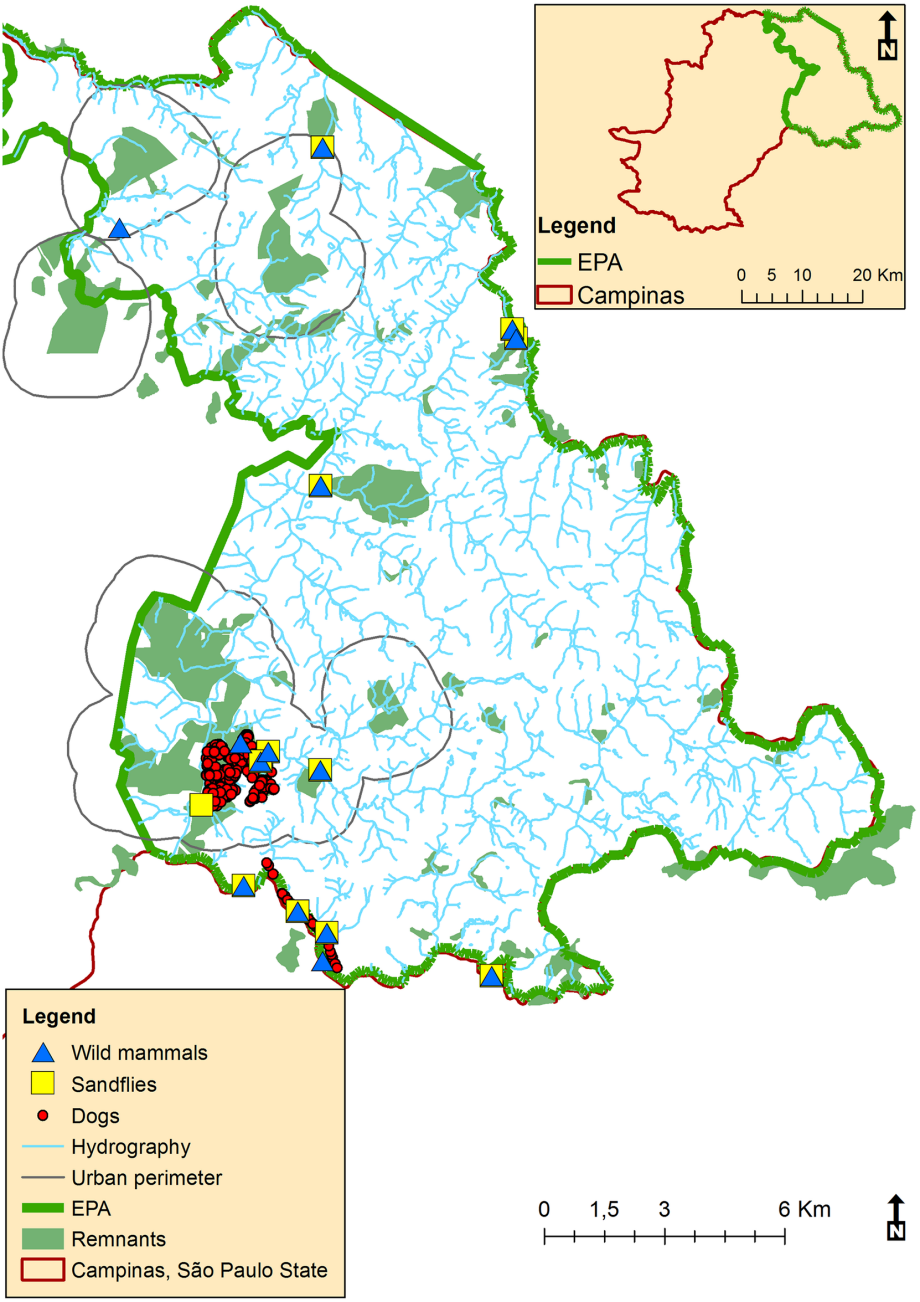
Location of the Campinas environmentally protected area (EPA), São Paulo, Brazil, indicating the sampling points of wild mammals and sand flies in the years of 2014 and 2015 and of dogs in 2013 and 2015.

A serological survey was conducted among domestic dogs in the condominium residences where the CVL focus was located. The sampling points, obtained by GPS (Global Position System), are shown in Fig 2.

The forest fragments and water resources of the EPA were mapped by the Municipal Health Department of Campinas using a geographic information system (GIS) with the MapInfo software, using a matrix layer of an aerial photograph of the region, obtained in 2007. A vector layer was superimposed, which showed the remnants of Atlantic Forest in the state of São Paulo, produced by the project “SOS Mata Atlântica”, obtained from the National Institute of Space Research website (INPE, http://www.inpe.br). ArcGIS software, v. 10.1 (Environmental System Research Institute, CA), was used to plot the geographic coordinates and draw the maps with all geographic objects and the cartographic base of the municipality available from the Brazilian Institute of Geography and Statistics (IBGE).

### Serological survey of domestic dogs

The survey area of canine cases in the EPA was defined based on the VL Surveillance Program of the state of São Paulo, which establishes a radius of 200 m from the confirmed canine case for conducting surveillance and control actions, and is expanded, whenever necessary, until samples from at least 100 dogs are obtained [21]. In this study, we included blood serum samples collected from all dogs resident in the two condominiums where the CVL focus was located, in addition to areas adjacent to the same (Fig 2).

The serological survey was conducted as part of the planned monitoring and control actions of the Municipal Health Department of Campinas and the IAL, performed in the dog population residing inside the EPA.

After authorization from the owners, data pertaining to the dogs were recorded in a database and serum samples were collected. The serological tests used were those recommended by the National Program for Surveillance and Control of Leishmaniasis: the Dual Path Platform rapid immunochromatographic test (TR DPP, Bio-Manguinhos, Brazil) for screening and the enzyme-linked immunosorbent assay (ELISA, Bio-Manguinhos, Brazil) to confirm the diagnosis, following the manufacturers’ protocols.

In this study, we included the surveys conducted in 2013 and 2015, considering that in 2014, there was no supply of kit for canine serological diagnosis from Brazilian Ministry of Health to the Municipal Health Department of Campinas, making it impossible to conduct the serological survey in this year, since this is the official technique for dog survey in Brazil.

### Sand fly and wild mammal collection

Sand flies and mammal captures were made monthly from April 2014 to March 2015, for three consecutive nights per month, simultaneously in three different points selected in EPA: one inside the condominiums where there is the CVL focus, another in the forest fragments with no contact with urban areas and a third randomly selected point.

For the collection of sand flies we used two or three modified CDC light traps (Horst, Brazil) per point of collection for two or three consecutive nights. The light traps were set up about one meter from the forest ground [22]. Species were classified as proposed by Galati [22] and the abbreviation scheme of genus names was that described by Marcondes [23].

Due to their high morphological resemblance, taxonomic identification of *Brumptomyia* females ended at the genus level. *Evandromyia sallesi* and *Ev*. *cortelezzii* were considered as *Ev*. *cortelezzii-sallesi*, based on the intermediate morphological forms observed and the morphological similarity of their females.

Wild mammals were captured for three consecutive nights per month, also from April 2014 to March 2015, using sixty tomahawk traps (Gabrisa, Brazil) with dimensions of 30 x 21 x 20 cm and 55 x 20 x 20 cm, baited with banana and chicken simultaneously at three different points in the EPA. A total of 20 traps per point (totally 60 traps) were distributed along the soil of forest region in linear transects with 10 to 20 meters between the traps [24].

The captured mammals were identified with microchip implants (Microchips Brasil, Brazil), and blood samples were collected by venipuncture during manual containment. Samples were stored at -20°C until DNA extraction.

The capture, containment, handling and sampling of the captured mammals were all carried out according to guidelines for the use of wild mammals in research, as recommended by the American Society of Mammologists [25].

### Genomic DNA extraction from samples of sand flies and wild mammals

DNA extraction of total blood samples from wild mammals was performed within 48 h of the conclusion of each capture, using a QIAamp DNA mini kit in a QIAcube DNA extractor (Qiagen, Netherlands).

To measure the quantity and purity of DNA from the samples, we used the NanoDrop ND-1000 (Thermo Fisher Scientific, EUA) equipment. To evaluate the endogenous quality, DNA integrity and the presence of inhibitors in blood samples, we performed PCR reactions using primers for the conserved gene of the interphotoreceptor retinoid-binding protein (IRBP), IRBP-CF-FWD (5'-TCCAACACCACCACTGAGATCTGGAC-3') and IRBP-CF-REV (5'-GTGAGGAAGAAATCGGACTGGCC-3') [26], or for the β1 (5’-ACCACCAACTTCATCCACGTTCACC-3’) and β2 genes (5’-CTTCTGACACAACTGTGTTCACTAGC-3’) [27].

The female sand flies were grouped in pools (1–6 specimens) according to species and location. DNA was extracted using a DNeasy Blood and Tissue kit (Qiagen, Netherlands). The quantity and purity of the DNA samples was measured using an Epoch spectrophotometer (BioTek, Winooski, Vermont).

### Detection of *Leishmania* DNA

PCR was performed using the primers LITSR (5’ CTGGATCATTTTCCGATG 3’) and L5.8S (5’ TGATACCACTTATCGCACTT 3’) to amplify a 300–350 base pair (bp) fragment from the intergenic region of the *Leishmania* DNA, internal transcribed spacer-1 (ITS-1) [28] and primers Lch14 and Lch15, which amplify a 167 bp of the kinetoplast minicircle DNA of *L*. (*L*.) *infantum* [29].

As reaction controls, we used ultrapure water and extracted DNA from *in vitro* cultures of standard strains of *L*. (*L*.) *infantum* (MHOM/BR/2002/LPC-RPV), *L*. (*V*.) *braziliensis* (MHOM/BR/1975/M2903) and *L*. (*L*.) *major* (MHOM/IL/1980/FRIEDLIN).

The reaction mixture contained 1.3 μL of buffer (50mM KCl, 20mM Tris-HCl pH 8.4), 0.4 μL MgCl2 (1.6 μM), 0.25 μL of each oligonucleotide (0.2 μM), 0.25 μL dNTP (0.2 mM), 0.25 μL of Platinum Taq DNA polymerase (Invitrogen, Brazil) and 8.3 μL of ultrapure water, with 1 μL of extracted DNA with a minimal concentration of 10 ng/μL. Thermal cycling conditions followed those of El Tai et al. (2000) [28] and Silva et al. [29]. The amplified products were identified by 1.5% agarose gel electrophoresis containing 1.0 μL/10 mL of SYBR safe DNA gel stain (Invitrogen, Life Technologies, USA).

### Genetic sequencing

Amplified products were purified with the Illustra GFX PCR DNA and Gel Band Purification kit (GE Healthcare, UK). Genetic sequencing by the Sanger method was performed in a Genetic Analyzer 3500 automated sequencer using a BigDye Terminator v 3.1 Cycle Sequencing kit (Applied Biosystems, Life Technologies, USA).

The sense and antisense sequences were visualized using Chromas v 2.1.1 software (Technelysium Pty Ltd, Australia), and were submitted to global alignment using MEGA5 software [30] and compared with sequences deposited in the GenBank, using the nucleotide basic local alignment search tool (BLASTn, http://www.ncbi.nlm.nih.gov/BLAST).

### Data analysis

The annual anti-*Leishmania* canine seroprevalences in the study area and the respective 95% confidence intervals (95%CI) were calculated. The frequency and percentages of sand flies and wild mammals infected, with their respective 95%CI, were also described. Statistical analysis was performed in Stata software, v. 11.0 (StataCorp LP, USA).

## Results

Human residences were observed in close proximity to the forest fragments, where the CVL focus is located inside the Campinas EPA (Fig 1). The distribution of trap sites for capturing sand flies and wild animals is shown in Fig 2, besides domestic dogs sampled.

**Fig 3:**
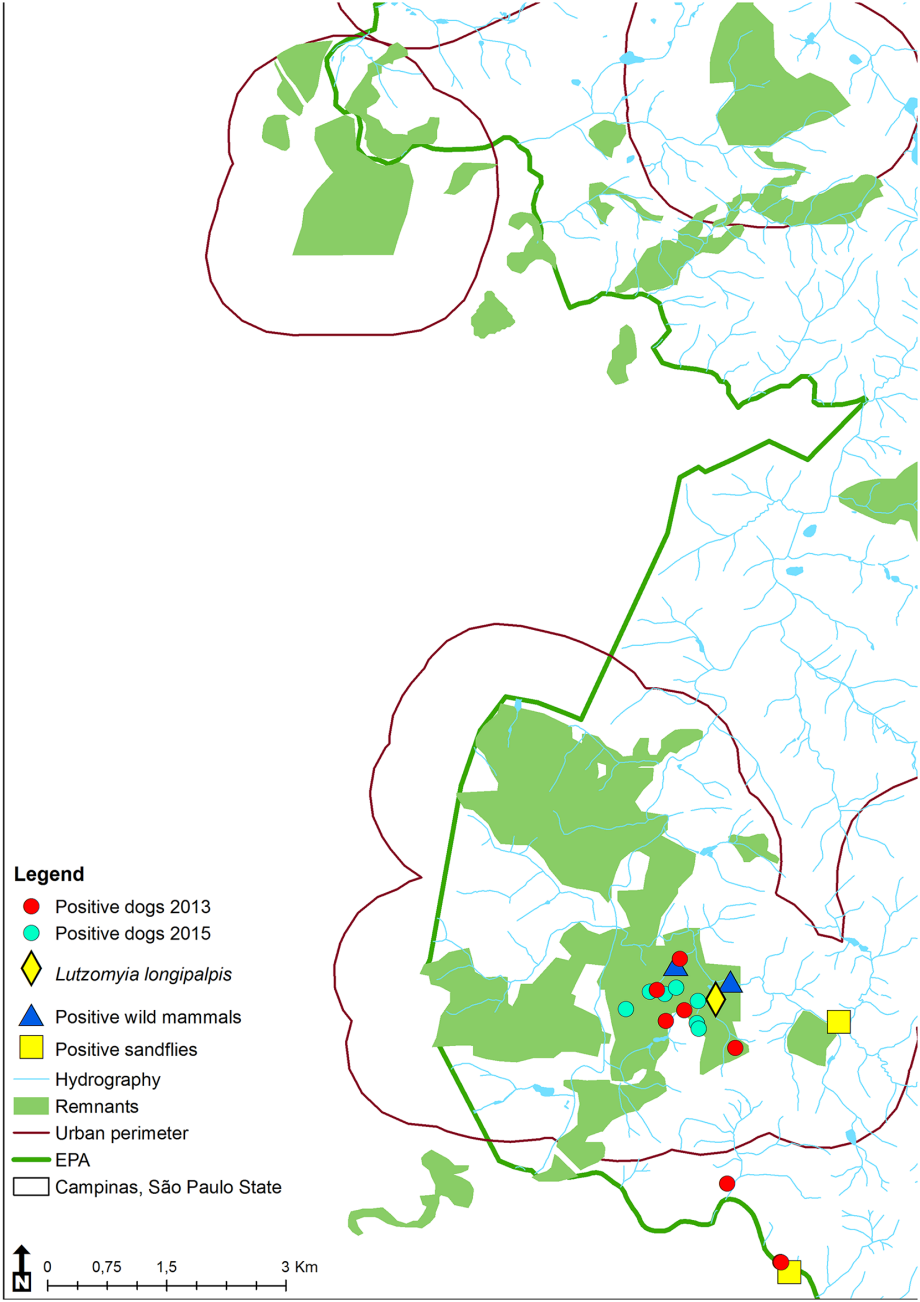
Campinas environmentally protected area (EPA), São Paulo, Brazil, showing the distribution of dogs positive for visceral leishmaniasis in 2013 and 2015 and of wild mammals and sand flies captured in 2014 and 2015 and naturally infected by *Leishmania* spp.

Fig 3 shows the spatial distribution of seropositive dogs, sand flies and wild mammals naturally infected by species of *Leishmania*, confirmed by molecular techniques.

### Serological surveys in dogs

In the canine surveys of 2013 and 2015 were evaluated, respectively, 590 and 571 blood samples from domestic dogs, performing 1,161 examinations (S1 Table). The seroprevalence determined and the respective confidence interval are shown in Table 1.

**Table 1:** Prevalence of anti-*Leishmania* antibodies determined in two canine serological surveys in the Campinas environmentally protected area, 2013 and 2015.

Year	No. of dogs tested	Reactive dogs^a^	Prevalence (%)	Confidence interval (95%)
**2013**	590	9	1.5	0.5–2.5
**2015**	571	7	1.2	0.3–2.1
**Total**	**1,161**	**16**	**1.4**	**0.7–2.0**

^a^Dogs that tested positive in screening with the Dual Path Platform rapid immunochromatographic test (TR DPP, Bio-Manguinhos, Brazil) and confirmation by enzyme-linked immunosorbent assay (ELISA, Bio-Manguinhos, Brazil). Data source: Zoonosis surveillance unit (UVZ), Municipal Secretariat of Health, Campinas, SP, Brazil.

### Phlebotomine sand flies

Four hundred and seventy-seven specimens of sand flies were collected, 291 of which were male and 186 female (S2 Table). Six species known or suspected of being vectors were identified as follows: *Nyssomyia whitmani* (107 males, 47 females), *Migonemyia migonei* (81 males, 34 females), *Pintomia fischeri* (21 males, 12 females), *Nyssomyia neivai* (12 males, 13 females), *Pintomyia pessoai* (3 males, 1 female) and *Lu*. *longipalpis* (2 males) (Table 2).

**Table 2:** Phlebotomine sand flies captured monthly using CDC light traps in the Campinas environmentally protected area, São Paulo, Brazil, from April 2014 to March 2015.

Species		Number of specimens			Relative % M/F
		Males	Females	N total	
***Brumptomyia* sp**.		52	43	95	19.92
***Evandromyia cortelezzii-sallesi***		3	2	5	1.05
***Evandromyia edwardsi***		0	1	1	0.21
***Evandromyia lenti***		0	1	1	0.21
***Expapillata firmatoi***		2	8	10	2.10
***Lutzomyia longipalpis***		2	0	2	0.42
***Migonemyia migonei***		81	34	115	24.11
***Nyssomyia neivai***		12	13	25	5.24
***Nyssomyia whitmani***		107	45	152	31.87
***Pintomyia fischeri***		21	12	33	6.92
***Pintomyia monticola***		2	23	25	5.24
***Pintomyia pessoai***		3	1	4	0.84
***Psathyromyia aragaoi***		0	1	1	0.21
***Psathyromyia pascalei***		5	2	7	1.47
***Psychodopygus ayrozai***		1	0	1	0.21
**Total**	Number	291	186	477	1.56
	**%**	**61.01**	**38.99**	-	**100**

PCR tests were performed on all 186 sand fly females, grouped in 74 pools (14 *Brumptomyia* sp., 2 *Evandromyia cortelezzii-sallesi*, 1 *Ev*. *edwardsi*, 1 *Ev*. *lenti*, 6 *Expapillata firmatoi*, 11 *Mg*. *migonei*, 3 *Ny*. *neivai*, 13 *Ny*. *whitmani*, 6 *Pi*. *fischeri*, 13 *Pi*. *monticola*, 1 *Pi*. *pessoai*, 1 *Psathyromyia aragaoi*, 2 *Pa*. *pascalei*), aimed at investigating the rate of natural infection.

The ITS-1 PCR detected a 300–350 bp fragment in three samples (1.6%; 95%CI 0.3–4.6%) belonging to *Ex*. *firmatoi* (1/10; 10.0%; 95%CI 0.2–44.5%) and *Pi*. *monticola* (2/25; 8.0%; 95%CI 1.0–26.0%) that characterized the samples as positive for *Leishmania* genus.

These three samples, one of *Ex*. *firmatoi* and two of *Pi*. *monticola*, were also amplified with Lch14/15 primers, producing the expected 167 bp products, later confirmed as *L*. *chagasi* (synonymous *L*. (*L*.) *infantum*) by genetic sequencing that showed 96 to 100% similarity with kinetoplast minicircle sequences deposited in the GenBank (Accession numbers AF308682.1, E values = 2e-37; 2e-50 and 2e-49).

### Wild mammals

Eighty-two wild mammals from six species were sampled: 1 *Mazama gouazoubira* (brocket deer; 1.2%), 1 *Sciurus* (*Guerlinguetus*) *aestuans* (squirrel; 1.2%), 8 *Callithrix jacchus* (white-tufted-ear marmoset; 9.8%), 11 *Didelphis aurita* (black-eared opossum; 13.4%), 18 *Callithrix penicillata* (black-tufted-ear marmoset; 22.0%) and 43 *Didelphis albiventris* (white-eared opossum; 52.4%). Six (6/82; 7.3%) *D*. *albiventris* were identified by a microchip reading in a second capture and sampled again (S3 Table).

Amplification of an expected 300–350 bp product was observed in 2/88 (2.3%; 95%CI 0.3–8.0%) samples from *D*. *albiventris* (2/43; 4.7%; 95%CI 0.6–15.8%). The first amplicon was confirmed as *L*. (*L*.) *infantum* and the second as *Leishmania* subgenus *Viannia*, making both the first records of infection by *Leishmania* species in wild mammals in this region [31].

In a third *D*. *albiventris* sample, a 500 bp product was confirmed as *Trypanosoma rangeli* by genetic sequencing (98% similarity; E value = 0.0, GenBank accession number AY230237.1 and others). No sample was positive when using the primers Lch14/15.

## Discussion

### Participation of wild fauna in transmission cycles of VL

The presence of natural infection by *L*. (*L*.) *infantum* in female sand flies and natural infection by two *Leishmania* species in a mammal species considered to be a potential reservoir (*D*. *albiventris*) provides strong evidence that sylvatic *Leishmania* transmission cycle is occurring in the EPA. However, it was not possible to confirm whether VL in Campinas occurred due to a wild enzootic cycle of transmission. Since dogs cohabit with infected wild animals, the hypothesis of the infection of wild mammals by infected dogs cannot be ruled out.

In the investigation conducted into the first case of CVL in the EPA in 2009, 40 wild mammals belonging to the species *Nectomys squamipes*, *D*. *albiventris*, *C*. *penicillata* and *Gracilianus agilis* were captured in wooded areas of the EPA and samples were examined by PCR, but none presented positive for VL [17]. In our investigation, infection among wild fauna by different species of *Leishmania* [31] was confirmed for the first time in this region, together with another species of trypanosomatid, *T*. *rangeli*. In addition, at the time of the first investigation, proximity of 100 m between human residences and forest areas where sand flies and wild mammals were captured was demonstrated [17].

In our study, although the capture of wild animals and phlebotominae was conducted in forest fragments in several locations within the EPA, the occurrence of *L*. (*L*.) *infantum* in these animals was observed only in locations close to seropositive domestic dogs. This spatial distribution suggests the involvement of these animals in the CVL focus, even though low *Lu*. *longipalpis* density and a small number of infected wild animals was observed.

Some authors suggest that the public health impact of VL infected dogs in urban areas is greater than in rural and wild regions, and that attention should be given to individuals living or frequenting these locations. Restricted contact between wildlife and domestic dogs has also been proposed to reduce the probability of VL transmission to humans and wild animals from dogs [32,33].

Discussion concerning the participation of wild species in the transmission of zoonotic parasites has become particularly important in recent years with the consolidation of the One Health concept [34,35]. Anthropogenic changes in ecosystems are particularly important in this context, resulting in greater proximity between wild and domestic animals and humans [36].

### Environmental degradation and proximity between wildlife and humans and their domestic animals

Proximity to forest areas and wildlife with human residences is a worrying scenario. In the state of Rio de Janeiro, dogs living within 100 m of forests presented a 3.5-fold higher risk of acquiring VL. The presence of opossums in the peridomicile increased the chances of infection in dogs 2.6-fold, with a 30.0% prevalence in these wild animals [37].

Another study detected a 20% prevalence of VL in dogs from rural areas in the state of Minas Gerais, located up to 2 Km from five EPAs that contained fragments of Atlantic Forest. The risk factors for these dogs were different from those living in urban areas [33].

In the Chaco region of Argentina, Barroso et al. [38] surveyed 77 dogs living in a forest area where two cases of human VL had occurred and determined a 13% prevalence. The authors suggested that the emergence of cases in dogs and humans in a wild region that was endemic for tegumentary leishmaniasis, with a relatively low canine prevalence, is clearly compatible with the involvement of wild mammals as reservoirs and that parasite transmission occurs from these animals. This scenario is very similar to the CVL focus located in the Campinas EPA.

### Sand fly fauna in the Campinas EPA

In the EPA, the presence of 15 phlebotomine sand flies species, even at low frequencies, shows a diversity of fauna, as previously reported in forest environments [39,40].

One interesting factor is the low density of *Lu*. *longipalpis* in the study area. Based on analysis of sexual pheromones secreted by males, it is accepted that *Lu*. *longipalpis* is a species complex [41]. The chemotype population of *Lu*. *longipalpis* found in Campinas, cembrene-1, is different to the chemotype population found in the western region of the state of São Paulo, (*S*)-9-methylgermacrene-B [42]. The remarkable differences between the epidemiological situations, population size and sibling complex of *Lu*. *longipalpis* in Campinas, corroborates the findings of Casanova et al. [43], who suggested there are different vectorial capacities and competence between siblings.

In this study, two species of phlebotomine sand flies, *Pi*. *monticola* and *Ex*. *firmatoi*, were found naturally infected with *L*. (*L*.) *infantum*. These species are essentially sylvatic and highly anthropophilic [44,45]. The participation of these species in the transmission of *L*. (*V*.) *braziliensis* and *L*. (*L*.) *infantum* has been suggested in the municipalities of Divinópolis, Minas Gerais, and in Rio de Janeiro, respectively, with *Pi*. *monticola* and *Ex*. *firmatoi* [46,47].

Females of different species were also found naturally infected by *L*. *(L*.*) infantum* in other endemic areas, such as specimens belonging to the *cortelezzii* complex in the Brazilian States of Minas Gerais and Mato Grosso do Sul—which are not amongst the incriminated leishmaniasis vector—and may be involved in a wild or rural cycle of *L*. (*L*.) *infantum* transmission in these areas [48,49]. *Mg*. *migonei*–a known vector of cutaneous leishmaniasis—was also found naturally infected in the Brazilian States of Pernambuco and Ceará. Some authors suggested that this species could act as a potential vector in VL transmission, particularly in areas where *Lu*. *longipalpis* is absent [50,51]. It is important to note that recent studies have demonstrated the high susceptibility of *Mg*. *migonei* to infection with *L*. (*L*.) *infantum*, reinforcing this hypothesis [52,53].

The detection of *Leishmania* DNA in a sand fly species does not prove vector competence [54]. We cannot exclude the persistence of DNA without any infective role, particularly because there are infected dogs and wild animals in the vicinity of these infected sand flies [55]. However, these results reinforce the need for further studies to investigate the vectorial capacity of these species, especially experimental studies.

### Particularities of VL focus in the study area

Although canine VL cases usually precede the occurrence of the disease in humans, no cases of human VL have been recorded in Campinas city to date. This fact could be associated with the local transmission characteristics related to the diversity and competence of the vectors, the presence of wild reservoirs and hosts, the characteristics of the parasite (not investigated), as well as the pattern of human contact, with transmission in sparsely populated area and in luxury condominiums.

The hypothesis that in some new VL foci in the state of São Paulo, subpopulations of *Lu*. *longipalpis* could be present and account for the existence and perpetuation of *L*. *(L*.*) infantum* enzootic wild cycles should be considered, particularly in areas around preserved forest environments or in recent residential projects.

On the other hand, Motoie et al. [56] identified the presence of genetically distinct populations of *L*. *(L*.*) infantum* in São Paulo, indicating that the inherent characteristics of the parasite itself could also be responsible for different epidemiological patterns of VL observed in new foci in the state.

In addition to factors directly associated with the transmission cycle, the occurrence of CVL cases in the Campinas EPA seems to be related to the occupation process, in which anthropic activities changed the natural landscapes, which has forest fragments containing wild fauna. In these regions, the exposure of humans and their pets to parasites and vectors of diseases to which they have not been previously exposed can result in disease outbreaks from natural enzootic foci.

The process of urban expansion within the EPA in the 1970s and 1980s was associated with an outbreak of cutaneous leishmaniasis in human inhabitants of the EPA in 1993 and 1994 [20]. In our study, an opossum was infected with a species of the subgenus *Viannia*, known to be responsible for tegumentary leishmaniasis in Brazil, which confirms the current risk of infection in this area and, again, the possible involvement of the wild fauna in the maintenance of the transmission cycle.

In addition, environmental modifications seem to be related to the adaptation of phlebotomine vectors to urban environments due to a decrease in the availability of wild animals as a food source, making dogs and humans more accessible alternatives to the vector [21,57].

### Particularities of different areas of VL transmission versus prophylaxis and disease control

The VL Surveillance and Control Program in Brazil supervises the main actions to reduce morbidity and lethality, aimed at early diagnosis and treatment of human cases, vector control and identification and elimination of seropositive domestic dogs [57]. Although there is good theoretical support for these measures, there is no evidence of their effectiveness in reducing the prevalence in endemic regions nor in the expansion of new outbreaks [58–60].

In Europe, although the domestic dog is considered the main reservoir in endemic areas, wild reservoirs have been proposed as potentially responsible for the lack of success in VL control [61]. In Brazil, the zoonotic transmission cycle is concentrated in areas where human habitation is located close to the wild cycle of the disease [2].

Although serological tests may be subject to false positive and/or negative results, due to its sensitivity and specificity, their use is valued for epidemiological surveillance purposes [22, 53].

Despite the serological tests used in this study, Grimaldi Jr. et al. [62]reported that DPP displayed high specificity (96%) but low sensitivity (47%) in identify asymptomatic dogs, but the sensitivity was significantly higher (98%) in diseased cases, indicating that this convenient test may be useful to identify the most infectious dogs.

In another study, Laurenti et al. [63] report that DPP detected both asymptomatic and symptomatic dogs in equal proportions. Using this test, 42/47 (89.4%) symptomatic dogs were detected positive, besides 35/38 (92.1%) asymptomatic dogs, with good accuracy of 92.7%. The ELISA BioManguinhos was positive in 43/47 (91.5%) symptomatic dogs and 34/38 (89.5%) asymptomatic dogs, with accuracy of 84.3%. Although, the combination of the two tests, as used in our study and in Brazilian surveillance activities, results in 99.1% sensitivity and 73.9% specificity, showing that it is a useful serology protocol for surveys.

In our study, the proximity of seropositive dogs housing sites with capture sites of wild mammals and sandflies infected with *Leishmania* spp. reinforces the assumption that they may be related. However, as shown in Fig 1, the human residences were constructed in the middle of the vegetation, being difficult to delimit the occurrence of independent transmission cycles in peridomiciliar or wild environment.

The need to elaborate distinct control and prophylaxis strategies, according to the characteristics of each transmission area, including the rural and wild areas in the control programs is now becoming evident. Studies that clarify the patterns of infection in different locations are useful for the success of such actions. Thus, investigating a new VL focus in all its distinct aspects contributes to our understanding of the key elements of the transmission dynamics and disease control. Actions that do not consider these particularities are less likely to succeed.

## Supporting information

S1 Table Data from domestic dogs studied in the transmission area of canine visceral leishmaniasis. Campinas Environmentally Protected Area, 2013 and 2015.
(XLSX)

S2 Table Data from sand flies studied. Campinas Environmentally Protected Area, April 2014 to March 2015.
(XLS)

S3 Table Data from wild mammals studied. Campinas Environmentally Protected Area, April 2014 to March 2015.
(XLS)

S1 Checklist For cross-sectional studies. (PDF)
