# Supplementary material for: Visceral leishmaniasis in an environmentally protected area in southeastern Brazil: Epidemiological and laboratory cross-sectional investigation of phlebotomine fauna, wild hosts and canine cases
Source: PLoS Negl Trop Dis. 2017 Jul 13;11(7):e0005666. doi: 10.1371/journal.pntd.0005666 (PMC5509102; doi:10.1371/journal.pntd.0005666)
Supplement: S1 Checklist — (PDF) [file pntd.0005666.s004.pdf]

STROBE Statement—Checklist of items that should be included in reports of ***cross-sectional studies***

	Item No	Recommendation
**Title and abstract**	1	(*a*) Indicate the study's design with a commonly used term in the title or the abstract<br><br>→ Visceral leishmaniosis in an environmentally protected area in southeastern Brazil: epidemiological and laboratory **cross-sectional** investigation of phlebotomine fauna, wild hosts and canine cases
		(*b*) Provide in the abstract an informative and balanced summary of what was done and what was found<br><br>→ Lines 36 to 48: "Methodology/Principal findings: We conducted a cross-sectional study of the VL focus to investigate *Leishmania* spp. infection in domestic dogs, wild mammals and sand flies using molecular tools and recommended serological techniques. Canine seroprevalences of 8.3%, 1.5% and 1.2% were observed in 2012, 2013 and 2015, respectively. Six insect species, confirmed or suspected vectors or potential transmitters of *Leishmania*, were identified. Two specimens of the main *L.* (*L.*) *infantum* vector in Brazil, *Lutzomyia longipalpis*, were captured in the study area. Natural infection by *L.* (*L.*) *infantum* was recorded in one *Expapillata firmatoi* specimen and two *Pintomyia monticola*. Natural infection by *L.* (*L.*) *infantum* and the *Leishmania* subgenus *Viannia* was also detected in two white-ear opossums (*Didelphis albiventris*), a known reservoir of VL. Geographical coordinates of each sampling of infected animals were plotted on a map of the environment protected area, demonstrating proximity between these animals, human residences, including the dogs positive for VL, and forest areas."
**Introduction**		
Background/rationale	2	Explain the scientific background and rationale for the investigation being reported<br><br>→ Lines 75 to 88: Background of visceral leishmaniosis (VL) in the last years, with the main epidemiology modifications, as it has emerged as an important public health problem in the last 35 years.<br>→ Lines 89 to 107: background of VL in Brazil and in the State of São Paulo. From 1980´s VL have occurred in urban environment with rapid expansion in several Brazilian municipalities.<br>→ Lines 108 to 116: background: environmental alterations versus VL expansion.<br>→ Lines 117 to 132: background of the study area, describing the epidemiological and environmental context in which the first VL case has occurred in a dog. The objectives of the study and the motivation of investigation was also described in these paragraphs.

Objectives	3	State specific objectives, including any prespecified hypotheses<br><br>→ Lines 129-132: "In order to identify the different components involved in the transmission cycle of this canine VL (CVL) focus in Campinas, we conducted a broad epidemiological investigation involving the capture of sand flies and free-living wild animals in other areas of the Environment Protected Area and integrated this research with the results of canine serological surveys."<br>We investigated if the new focus of CVL in Campinas had the participation of wild species.
**Methods**		
Study design	4	Present key elements of study design early in the paper<br>→ The key element of this study is the integration of several aspects of the investigation of the CVL focus through research on vector infection, wild mammals, domestic dogs and environmental characteristics of the study area.
Setting	5	Describe the setting, locations, and relevant dates, including periods of recruitment, exposure, follow-up, and data collection<br>→ Lines 136 to 179: The study was conducted in an Environment Protected Area in a large city in Southeastern Brazil, where a focus of CVL was detected in 2009. The area contains many forests fragments with wild species, and human residences nearby. In April 2015 to March 2016, we monthly (3 nights each) captured sand-flies (477) and wild mammals (82) and collected blood samples from the latter for molecular techniques. Dogs were tested thorough serology anti Leishmania in three surveys, 2012, 2013 and 2015.<br>→ Lines 182 to 191: These paragraphs describe the serological investigation in domestic dogs, including the description of the locations and dates.<br>→ Lines 201 to 208: These paragraphs describe the capture of sand flies, including the description of the locations and dates.<br>→ Lines 213 to 218: This paragraph describes the capture of wild mammals, including the description of the locations and dates.
Participants	6	(*a*) Give the eligibility criteria, and the sources and methods of selection of participants<br>→ Lines 182 to 188: describes the methods of selection of domestic dogs – "The survey area of canine cases in the EPA was defined based on the VL Surveillance Program of the state of São Paulo, which establishes a radius of 200 m from the confirmed canine case for conducting surveillance and control actions, and is expanded, whenever necessary, until samples from at least 100 dogs are obtained [22]. For this study, however, blood serum samples were collected from all dogs resident in the two condominiums where the CVL focus was located, in addition to adjacent areas (Fig 2)."<br>→ Lines 201 to 208 and 213 to 218: these paragraphs describe the capture of sand flies and wild mammals.<br>→ We examined and tested all wild mammals and sandflies captured.

Variables	7	Clearly define all outcomes, exposures, predictors, potential confounders, and effect modifiers.<br>→ Our objective was to estimate the prevalence of *Leishmania* ssp infection in wild mammals, sand flies, and dogs domiciled in the study area.<br>Give diagnostic criteria, if applicable<br>→ Lines 192 to 198: Diagnostic criteria to determine seropositivity in domestic dogs was described.<br>→ Lines 245 to 261: Diagnostic criteria to determine *Leishmania* spp. And *Leishmania* (*L.*) *infantum* infection by molecular tools was described.
Data sources/ measurement	8*	For each variable of interest, give sources of data and details of methods of assessment (measurement). Describe comparability of assessment methods if there is more than one group<br>→ Not applicable
Bias	9	Describe any efforts to address potential sources of bias<br>→ Serological investigation of domestic dogs: we only consider as positive the dogs that presented reactive results in the two official techniques of serological diagnosis of VL in Brazil, recommended by the Ministry of Health.<br>→ Molecular investigation of wild mammals and sand flies: in the application of molecular techniques, we were cautions in confirming the identity of amplicons by genetic sequencing. In addition, we ensured sufficient DNA concentration in samples tested.
Study size	10	Explain how the study size was arrived at<br>→ We studied domestic dogs in a geographically restricted focus of CVL and all wild mammals and sand flies captured in a period of 12 months.
Quantitative variables	11	Explain how quantitative variables were handled in the analyses. If applicable, describe which groupings were chosen and why<br>→ We presented prevalence (%) with the respective confidence intervals 95% of species of sand flies and wild mammals captured and the prevalence of positive serology among dogs in each year (with the respective confidence intervals 95%).
Statistical methods	12	(*a*) Describe all statistical methods, including those used to control for confounding<br>→ We did not use statistical methods because it is a cross-sectional descriptive study. We only access percentages and its respective 95% confidence intervals of animals infected by *Leishmania* spp. This information was described in Lines 283 to 287: “The annual anti-*Leishmania* canine seroprevalence in the study area and the respective 95% confidence intervals (95%CI) were calculated. The frequency and percentages of sand flies and wild mammals infected, with their respective 95%CI, were also described. The percentages and 95%CI were calculated using Stata software, v. 11.0 (StataCorp LP, USA).”
		(*b*) Describe any methods used to examine subgroups and interactions

		→ not applicable
		(*c*) Explain how missing data were addressed<br>→ not applicable
		(*d*) If applicable, describe analytical methods taking account of sampling strategy<br>→ Because we didn't have information about the total population of sand flie and wild mammal fauna and not even about the prevalence of *Leishmania* infection in these animals, we didn't apply any calculation for the sample design. We used strategies for designing the captures of these animals, which were described in lines 161 to 179 and 201 to 208.<br>→ Sampling strategy for domestic dogs was described in lines 182 to 193.
		(*e*) Describe any sensitivity analyses<br>→ not applicable
**Results**		
Participants	13*	(a) Report numbers of individuals at each stage of study—eg numbers potentially eligible, examined for eligibility, confirmed eligible, included in the study, completing follow-up, and analysed<br>→ Lines 303 to 305 and S1 Table: The total number of examinations of domestic dogs (1378) in the three years studied were described.<br>→ Lines 319 to 324, S2 Table and Table 2: Describe the information about all the sand flies (477) individuals studied.<br>→ Lines 345 to 351 and S3 Table: Describe the information about each wild mammal (82) captured and studied.
		(b) Give reasons for non-participation at each stage<br>→ not applicable
		(c) Consider use of a flow diagram<br>→ not applicable
Descriptive data	14*	(a) Give characteristics of study participants (eg demographic, clinical, social) and information on exposures and potential confounders<br>→ We examined, classified and tested all 82 wild mammals and 477 sand flies captured and plotted each location on the map (Fig. 2). We also plotted the geographic coordinates (X, Y) of all positive dogs tested by serology on the map of the EPA (Fig. 3). The collected data regarding each of the studied animals are described in the three tables of supporting information (S1, S2 and S3 Table).
		(b) Indicate number of participants with missing data for each variable of interest<br>→ not applicable
Outcome data	15*	Report numbers of outcome events or summary measures<br>→ The main outcome data were:<br>Two specimens of the main *L.* (*L.*) *infantum* vector in Brazil, *Lutzomyia longipalpis*, were captured in the study area; Natural infection by *L.* (*L.*) *infantum* was recorded for the first time in these CVL focus in one *Expapillata firmatoi* specimen and two *Pintomyia monticola*; Natural infection by *L.* (*L.*) *infantum* and the

		Leishmania subgenus *Viannia* was also detected in two white-ear opossums (*Didelphis albiventris*), a known reservoir of VL, also for the first time in these CVL focus.
Main results	16	(*a*) Give unadjusted estimates and, if applicable, confounder-adjusted estimates and their precision (eg, 95% confidence interval). Make clear which confounders were adjusted for and why they were included<br>→ not applicable
		(*b*) Report category boundaries when continuous variables were categorized<br>→ not applicable
		(*c*) If relevant, consider translating estimates of relative risk into absolute risk for a meaningful time period<br>→ not applicable
Other analyses	17	Report other analyses done—eg analyses of subgroups and interactions, and sensitivity analyses<br>→ We built a map (Fig 3) with all locations of positive results in dogs, sand flies, and wild mammals, considering their proximity to forest and urbanized spots.
**Discussion**		
Key results	18	Summarise key results with reference to study objectives<br>→ Spatial distribution of infected mammal with L. (L.) *infantum*, seropositive domestic dogs and the presence of phlebotomine sand flies suggest involvement of wild fauna in CVL, and it seems to be related to anthropic activities in the area.
Limitations	19	Discuss limitations of the study, taking into account sources of potential bias or imprecision. Discuss both direction and magnitude of any potential bias<br>→One limitations of the study is the number of catches of wild vectors and mammals. Probably a larger sample and in wider distribution could yield more consistent results on the participation of the wild cycle in the occurrence of the focus of CVL. Besides that, the possibility of false-positive or false-negative results in the serological techniques used for dogs cannot be ruled out.
Interpretation	20	Give a cautious overall interpretation of results considering objectives, limitations, multiplicity of analyses, results from similar studies, and other relevant evidence<br>→ Local transmission characteristics, as sparse population, low density of *Lu. longipalpis,* other phlebotomine species with infection by *Leishmania* (*L.*) *infantum*, no human cases after canine transmission, occupation of forests by human habitation, presence of wild animals with parasitaemia by pathogenic species of *Leishmania*, require specific actions and routine review of disease prevention and control, especially in Brazil, where the current control program was developed decades ago.
Generalisability	21	Discuss the generalisability (external validity) of the study results<br>→Discussion concerning the participation of wild life species in the transmission of zoonotic parasites became particularly important in the context of the consolidation of One Health concept.

		→ Anthropogenic changes in ecosystems may result in proximity between wild and domestic animals and humans and in alterations on the fauna dynamic, which may be expose animals and/or humans to pathogens.<br>→ Surveillance and control programs of VL should be reviewed periodically and consider the particularities of each area, including its environmental and occupancy characteristics.<br>→ Investigation of new species of mammals and vectors involved in each transmission cycle of VL or cutaneous leishmaniosis are very important to establish successful control actions.
**Other information**		
Funding	22	Give the source of funding and the role of the funders for the present study and, if applicable, for the original study on which the present article is based<br>→ According to the submission guidelines of PloS NTD, funding sources should not be cited in the body of the text, but in a specific field in the online submission system. The follow information will be include at the moment of submission: "This research received financial support of São Paulo Research Foundation (FAPESP, http://www.fapesp.br), grant nos. 2014/27212-0, 2014/13049-0 and 2016/02572-0, besides the financial support of the Research Program for the Unified Health System (PPSUS, FAPESP, CNPq—National Council for Scientific and Technological Development; http://portalsaude.saude.gov.br/index.php/o-ministerio/principal/secretarias/sctie/ppsus) No. 12/51267-4.The funders had no role in study design, data collection and analysis, decision to publish, or preparation of the manuscript."

*Give information separately for exposed and unexposed groups.

Note: An Explanation and Elaboration article discusses each checklist item and gives methodological background and published examples of transparent reporting. The STROBE checklist is best used in conjunction with this article (freely available on the Web sites of PLoS Medicine at http://www.plosmedicine.org/, Annals of Internal Medicine at http://www.annals.org/, and Epidemiology at http://www.epidem.com/). Information on the STROBE Initiative is available at www.strobe-statement.org.
